# Smartphone-based mobile applications for adverse drug reactions reporting: global status and country experience

**DOI:** 10.1186/s12911-022-01832-7

**Published:** 2022-05-02

**Authors:** Ayako Fukushima, Noha Iessa, Madhava Ram Balakrishnan, Shanthi Narayan Pal

**Affiliations:** Regulation and Safety, World Health Organization, Avenue Appia 20, 1211 Geneva, Switzerland

**Keywords:** Smartphone apps, Adverse drug reactions (ADRs), Drug safety, Reporting of ADRs, VigiBase, Regulatory system, Pharmacovigilance

## Abstract

**Background:**

Smartphone technology can support paperless reporting of adverse drug reactions (ADRs). The aims of this study were to systematically assess smartphone ADR-reporting applications, understand their qualitative and quantitative impact on ADR reporting, and garner key lessons from owners and developers.

**Methods:**

This study had three components: (1) An assessment of ADR-reporting apps, (2) an online survey on the impact of app implementation on ADR reporting and the experiences of app developers and owners, and (3) a search of VigiBase, the World Health Organization global database of individual case safety reports (ICSRs), to observe trends in the number of ADR reports targeting countries where the apps were implemented.

**Results:**

Twenty-two apps were included. Eight out of the 22 apps were for countries in the WHO African region. Features observed included E2B data elements (E stands for efficacy) and functions supporting reporting and user engagement. Seventeen app developers and owners answered to the survey and reported overall positive experiences with app features, and post-launch increases in the total number of ICSRs. User type and user environment were cited as factors influencing app use: Respondents said younger people and/or those with an inclination to use technology were more likely to use apps compared to older or more technology-averse people, while respondents in countries with limited internet connectivity reported persistent difficulties in app use.

**Conclusions:**

Smartphone apps for reporting ADRs offer added value compared to conventional reporting tools. Reporting tools should be selected based on interface features and factors that may influence app usage.

## Background

Spontaneous reporting of adverse drug reactions (ADRs), defined here as unsolicited communication by a reporter to a competent authority, such as a national regulatory authority (NRA), has been the conventional approach to pharmacovigilance notification since the early 1960s [1, 2]. Many studies have been conducted to identify potential factors affecting reporting behavior among different types of reporters [3–5], and various approaches have been undertaken to promote pharmacovigilance and to encourage reporting [6–8].

An average of 2 million individual case safety reports (ICSRs) are recently added each year to the World Health Organization’s global database of ICSRs, VigiBase, from more than 100 countries. By the end of 2020, the total number of ICSRs had exceeded 24 million [9] since VigiBase’s establishment in 1968. The E2B format (E stands for efficacy) defined by the International Council for Harmonisation of Technical Requirements for Pharmaceuticals for Human Use (ICH) has helped standardize the data elements, structure and formats of ICSRs regardless of source or destination [10], ultimately contributing to the overall improvement in the quality of reports in the global database.

However, despite this fundamental improvement, spontaneous reporting has been significantly limited by under-reporting, poorly documented reporting and reporting delays. These challenges were due to a lack of knowledge and awareness of pharmacovigilance and a lack of motivation, as well as indifference, insecurity, complacency, workload, and lack of training [11, 12]. The lack of prompt, well-documented reports hampers NRAs from making timely public health decisions for the safer use of medicinal products [13].

Paper-based reporting has long been the primary method for collecting ADRs [14]. However, to improve ADR reporting, paperless approaches have been explored, including via text message [15], telephone [16], websites (e-reporting) [17] and social media [18]. Furthermore, we are seeing the use of smartphone-based tools [19, 20]. Such tools also exist in a variety of other healthcare areas, such as disease management, drug dictionaries and drug dosage calculators [21, 22].

Mobile phone applications (apps) designed for ADR reporting have been attracting the attention of NRAs, health care professionals (HCPs) and patients, not only from high-income countries (HICs) but also from low- and middle-income countries (LMICs) [23–27]. Since 2017, The World Health Organization (WHO) has been supporting LMICs to roll out the ADR reporting app, Med Safety, in collaboration with technical partners, including the United Kingdom Medicines and Healthcare products Regulatory Agency (MHRA) and the Uppsala Monitoring Centre (UMC). The focus has been on improving the quality and quantity of ADR reports within countries in real time, reducing the burden of reporting and managing those reports, and improving user engagement by providing post-reporting feedback and drug safety information.

While there is a strong interest among NRAs, HCPs and patients in transitioning to app-based ADR reporting, little was known about existing ADR reporting apps, their characteristics and performance. This study aimed at systematically assessing and characterizing accessible smartphone ADR reporting apps, to understand the impact of their use on the quality and quantity of ADR reporting, and to collate lessons learned from the experiences of developers and owners.

## Methods

This study consisted of three components: (1) A systematic assessment of ADR reporting apps identified through an extensive search of smartphone app stores and the internet, (2) an online survey targeting developers and owners on the impact of app implementation on ADR reporting and their experiences of the identified apps and (3) a quantitative search of VigiBase to observe trends in the number of ADR reports in countries where the identified apps were implemented. We defined “developers” as entities that technically develop mobile apps, “owners” as entities that oversee the apps and for whom the apps are developed (e.g., NRAs), and “users” as individuals who use the apps to report ADRs or access drug safety information.

### Systematic assessment of ADR reporting apps available worldwide

The Preferred Reporting Items for Systematic reviews and Meta-Analyses (PRISMA) checklist was consulted to draw up an evidence-based minimum set of items required for reporting in systematic reviews [28]. This checklist provides for transparent reporting of a systematic review of scholarly references. Since this study targeted smartphone apps instead of scholarly references, we conducted it using deviations from the original checklist of items to include when reporting a systematic review when it came to bias risk-related items in individual studies and across studies (items 12, 15, 19 and 22) [28].

#### Eligibility

Inclusion criteria: An “app” was defined as a software application designed to run on a smartphone, and was included in the study if it permitted users to directly create an ADR report within the app and submit it to a given addressee. Apps in any language were eligible.

Exclusion criteria: Apps without any automated ADR report submission or provision of appropriate addressees were excluded. Apps that only focused on reporting events on items unrelated to medicines, such as cosmetic products, medical devices and vaccines, were also excluded. App price was not included in the selection criteria.

#### Information sources and search strategy

A search of the App Store and the Google Play Store was conducted using 17 search terms to identify potentially eligible ADR reporting apps. The search terms were reviewed and validated to ensure their relevance and exhaustivity. The search terms are listed in “Appendix 1”.

The App Store is a digital distribution service developed by Apple (Apple Inc., United States of America) and serves as the official app store of devices using the iOS mobile operating system. The Google Play Store is a service developed by Google (Google, LLC, United States of America) and serves devices using the Android system. Other operating systems were not considered in this study given that, as on July 2020, more than 99% of the global market share of mobile operating systems was covered by either Android or iOS [29] and the search on both the App Store and the Google Play was assumed to be comprehensive enough to generalize to the global setting.

In general, search results retrieved by an internet search engine are personalized based on search history and user location. Logging out of an account or searching through an incognito window were ensured to get neutral search results that were not influenced by personalized information [30].

The search using the defined search terms in both smartphone app stores was performed on August 19, 2020. Search results were exported and saved in two Excel spreadsheets. The following information was collected in the spreadsheets: name of app, name of developer or owner, specific URL in each mobile app store and content type. Deduplication of apps was performed using the URL information.

In addition, an internet search for apps satisfying the selection criteria was conducted using an incognito window in Chrome and Firefox browsers. The same search terms as used in the app stores were applied. This desk research was performed in November 2020.

Each search was performed in Geneva, Switzerland.

#### Selection process

A two-step process was adopted to select the apps for inclusion in the study. The first consisted of reviewing the name of the app and its descriptions available in the app stores to determine whether it met the selection criteria. The second consisted of downloading and installing the apps on either an Android phone (Xiaomi Mi9T) or Apple iPhone (iPhone 8) depending on the source. A full review was then performed of the entire app. The PRISMA flow diagram was created using open-source software draw.io to illustrate the selection process [31].

#### Data extraction process

The following information was collected and extracted using Excel software:

- General information on each app, such its name, developer or owner name, available platform, app version tested, size, cost per installation and use, and the latest update.
- Geographic scope of where each app could submit reports. To categorize the countries, we used the six WHO regions, namely African Region, Region of the Americas, South-East Asia Region, European Region, Eastern Mediterranean Region, and Western Pacific Region, and the definition of the World Bank income groups, namely low, lower-middle, upper-middle, and high [32].
- The features of each app (e.g., offline report development, supplementary materials attachment and field for laboratory test results, as well as two-way communication-related features).
- The E2B data elements available in each app. The most frequently available data elements were identified during a pilot assessment of multiple apps. The identified data elements were explored during the data extraction process and stored under a category of the “minimum information for valid safety report” as defined by the ICH: Identifiable patient, identifiable reporter, adverse event/reaction, and suspect or interacting drug [10]).

Apps available only in languages other than English were translated into English during data extraction.

### Online survey

The online survey was designed to interview developers and owners of the selected apps to obtain information on the impact of app implementation on the quality and quantity of ADR reports, and to understand their experience of the apps. The online survey was created using Google Forms and an invitation was emailed to the developers and owners of the selected apps. The online survey targeted pharmacovigilance officers in the National Regulatory Agencies, academia and pharmaceutical industry to ensure reliable information and subject knowledge when responding to the survey queries. Up to two reminders were sent to those who did not respond. The survey consisted of 9 open-ended and 10 closed questions (“Appendix 2”).

The apps’ quantitative impact was assessed using responses to questions on total number of downloads, trends in the overall number of ADR reports after the app launch, total number of ADR reports received from the app, and proportion of ADR reports received from the app among all reports since the launch. The total numbers of ADR reports received from the app were also averaged by year based on the number of years from app launch to the survey. The collected responses on the number of downloads of different versions of the Med Safety app were confirmed by MHRA, which had access to such information.

The apps’ qualitative impact was assessed using responses to questions on the availability of the minimum four information items for a valid safety report as defined by the ICH: Identifiable patient, identifiable reporter, adverse event/reaction, and suspect or interacting drug [10]. The respondents were invited to select those of the four that were judged to have been appropriately filled in by the reporters of the ADR reports received through paper forms and apps.

The lessons learned from the respondents’ experiences of the app were sought through open-ended questions. The feedback was reviewed and synthesized into the following themes: simplicity of use, report quality, accessibility, innovativeness, data transferability and data sharing, two-way communication, cost, and data security.

Additional information—such as the app’s launch date, the purpose of its implementation, and its development and maintenance costs—was also obtained.

### Quantitative search of number of reports submitted to VigiBase before and after app launch

VigiBase was searched to observe trends in the number of ADR reports submitted before and after an app’s launch. VigiLyze, the analytical platform of VigiBase, was used for this analysis. VigiLyze did not allow searches by type of reporting tool. Thus, only the overall number of ADR reports, regardless of type of reporting tool, was extracted according to the following search conditions:

- Geographical scope: Countries where the selected apps were implemented.
- Timeframe:
  - The pre-launch period was defined as the 12 months preceding the app’s launch.
  - Post-launch Period A was defined as the period from the launch to Month 12 after the launch.
  - Post-launch Period B was defined as the period from Month 13 to Month 24 after the launch.

Although the numbers of ADR reports were not analyzed by type of reporting tool, this analysis was considered to illustrate the quantitative impact of app implementation in the countries.

The VigiBase search was performed on February 15, 2021. Only apps that had already been launched for at least one year (12 months) by that date were included in the analysis, as well as those that had been launched for at least two years (24 months) so that, at a minimum, data for Period A was available, and in some cases also data for Period B. Since the launch date was required to set the timeframes to be analyzed, an app was only included if the online survey response supplied its launch date. Relative percentage changes were calculated to compare the numbers of ADR reports received in post-launch Periods A and B with the number in the pre-launch period for each app.

## Results

### Search results and app inclusion

**Fig. 1:**
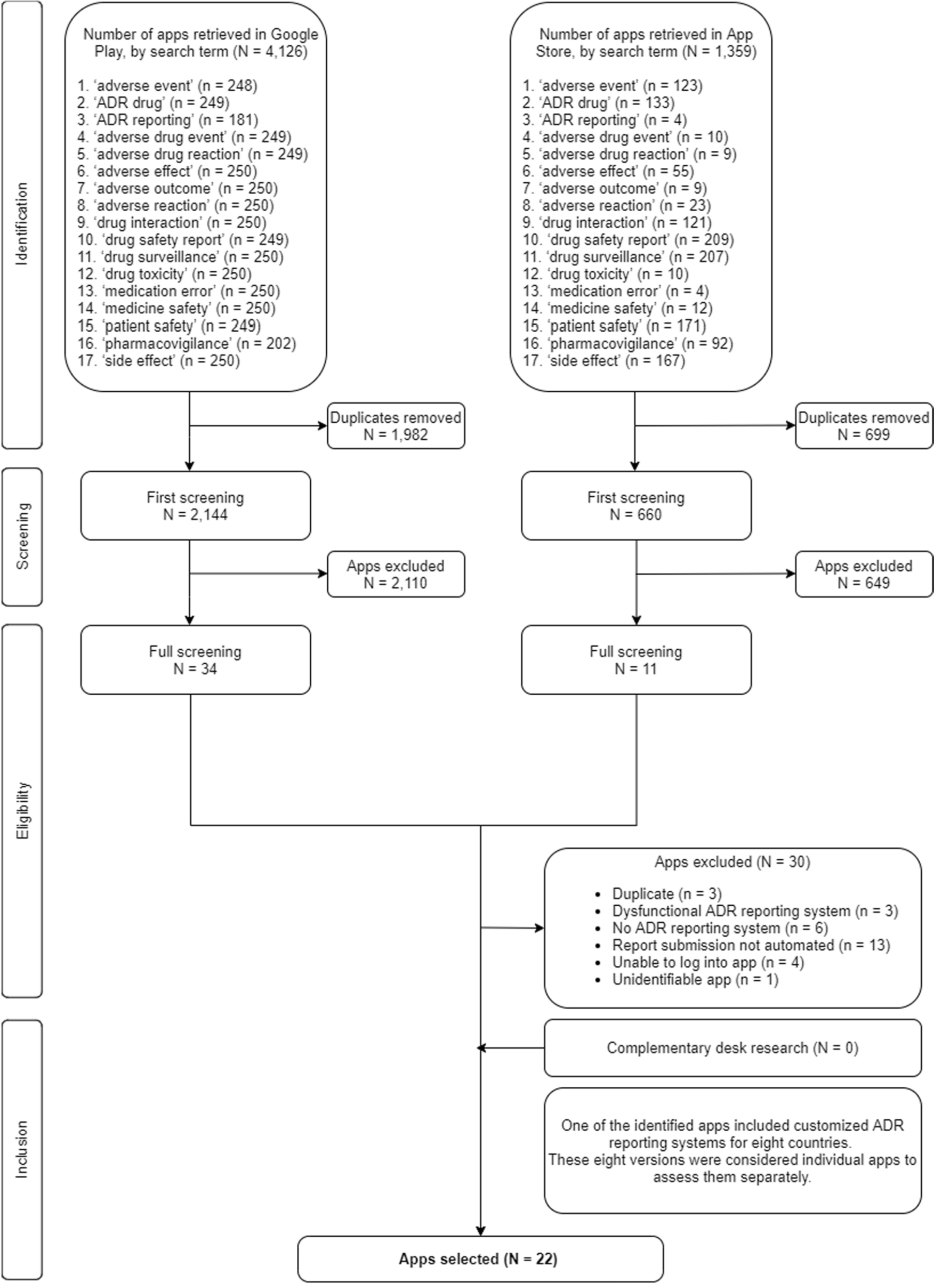
PRISMA flow chart of the app selection process

A flowchart of the app selection is shown in Fig. 1. The searches in the Google Play Store and App Store resulted in the retrieval of 4126 and 1359 apps, respectively. The number of hits varied depending on the search terms, from 181 hits for ‘ADR reporting’ to 250 hits for each of nine other terms in the Google Play Store, and from four hits for ‘ADR reporting’ to 209 hits for ‘drug safety report’ in the App Store. After removing 2681 duplicates, 2144 and 660 apps, from each store respectively, were included in the first screening stage during which the names and descriptions of apps were reviewed. Following the first screening stage, 2110 apps retrieved from the Google Play Store and 649 apps from the App Store were excluded as they did not meet the selection criteria. This resulted in the retrieval of 34 apps from the Google Play Store and 11 apps from the App Store for the second screening stage (full screening). Thirty apps were excluded in the full screening for the following reasons: “Duplicate” (n = 3), “Dysfunctional ADR reporting system” (n = 3), “No ADR reporting system” (n = 6), “Report submission not automated” (n = 13), “Unable to log into app” (n = 4) and “Unidentifiable app”^1^ (n = 1). No additional apps were identified during the desk research using the Chrome and Firefox browsers.

One of the identified reporting apps, Med Safety, had several versions, each adapted to the ADR reporting system of each of eight countries. To assess the versions separately and to facilitate the data extraction process, each of the eight was considered an individual app. Consequently, the final number of apps that met the inclusion criteria was 22.

#### Basic and geographic specifications

The basic specifications of the 22 selected apps are listed in Table 1. No app required payment by users for download and use. The selected apps were developed by 12 developers, and the MHRA was cited as a developer for 11 apps, such as HALMED, Med Safety, UAE RADR and Yellow Card Scheme. Six of the 22 apps were available only on Android, one only on iOS, and 15 on both platforms. The last update occurred in the study year (i.e. 2020) for 14 apps, one year before the study year (2019) for three apps, two years before (2018) for two apps, three years before (2017) for two apps, and four years before (2016) for one app.

**Table 1:** Technological specifications of the selected apps

App number	App name	Platform	Developer	Tested app version	App size	Last update
1	ADR Online	iOS ^URL1^	Simon Watt	2.0.1	5.6 MB	1 November 2018
2	ADR PvPI	Android ^URL2^	Pharmacovigilance Programme of India (PvPI)	1.8.0	4.8 MB	14 July 2020
3	ADR Reporter	Android ^URL3^	SYED SHARIQ NAEEM	1.0	0.368 MB	16 October 2016
4	DGDA Drug Verification	Android ^URL4^	Access to Information Programme	2.2	5.4 MB	1 July 2019
5	Easypharm	Android ^URL5^, iOS ^URL6^	Android: PGE2 sprl iOS: PGE2	1.1.22	Android: 7.6 MB iOS: 21.4 MB	Android: 8 November 2017 iOS: 13 November 2017
6	ELEA Onco-Biotech	Android ^URL7^, iOS ^URL8^	Laboratorio Elea	1.0	Android: 19 MB iOS: 58.1 MB	23 December 2017
7	HALMED	Android ^URL9^, iOS ^URL10^	Medicines & Healthcare products Regulatory Agency	22.0.0	Android: 16 MB iOS: 16.6 MB	30 October 2020
8	Med Safety (Armenia)	Android ^URL11^, iOS ^URL12^	Android: WEB-RADR iOS: MHRA	22.0.0	Android: 16 MB iOS: 17 MB	26 October 2020
9	Med Safety (Botswana)	Android ^URL11^, iOS ^URL12^	Android: WEB-RADR iOS: MHRA	22.0.0	Android: 16 MB iOS: 17 MB	30 October 2020
10	Med Safety (Burkina Faso)	Android ^URL11^, iOS ^URL12^	Android: WEB-RADR iOS: MHRA	22.0.0	Android: 16 MB iOS: 17 MB	31 October 2020
11	Med Safety (Côte d’Ivoire)	Android ^URL11^, iOS ^URL12^	Android: WEB-RADR iOS: MHRA	22.0.0	Android: 16 MB iOS: 17 MB	22 October 2020
12	Med Safety (Ethiopia)	Android ^URL11^, iOS ^URL12^	Android: WEB-RADR iOS: MHRA	22.0.0	Android: 16 MB iOS: 17 MB	July 2020
13	Med Safety (Ghana)	Android ^URL11^, iOS ^URL12^	Android: WEB-RADR iOS: MHRA	22.0.0	Android: 16 MB iOS: 17 MB	17 November 2020
14	Med Safety (Uganda)	Android ^URL11^, iOS ^URL12^	Android: WEB-RADR iOS: MHRA	22.0.0	Android: 16 MB iOS: 17 MB	30 October 2020
15	Med Safety (Zambia)	Android ^URL11^, iOS ^URL12^	Android: WEB-RADR iOS: MHRA	22.0.0	Android: 16 MB iOS: 17 MB	2 November 2020
16		Android ^URL13^, iOS ^URL14^		2.3	Android: 24 MB iOS: 48.8 MB	15 November 2019
17	My eReport	Android ^URL15^, iOS ^URL16^	eVeDrug	Android: 1.12 iOS: 2.9.3	Android: 4.3 MB iOS: 9.3 MB	16 January 2018
18		Android ^URL17^	ysbda-dev	1.0.1	4.2 MB	21 June 2020
19	SiddAR	Android ^URL18^	SCRI PHARMACY, CCRS, MoAYUSH, GoI	2.0	4.5 MB	03 October 2019
20	TMDA Adverse Reactions Reporting Tool	Android ^URL19^	Hakiki Dawa	1.2.0	7.5 MB	29 June 2020
21	UAE RADR	Android ^URL20^, iOS ^URL21^	Android: Medicines & Healthcare products Regulatory Agency iOS: MHRA	22.0.0	Android: 16 MB iOS: 17.4 MB	1 November 2020
22	Yellow Card Scheme^a^	Android ^URL22^, iOS ^URL23^	Android: Medicines & Healthcare products Regulatory Agency iOS: MHRA	22.0.0	Android: 16 MB iOS: 17.9 MB	3 November 2020

Where data is different between Android and iOS, they are shown separately for each platform. When the update dates retrieved from the website conflicted with those in the questionnaire, the answer from the questionnaire was prioritized

^a^The same app was named Yellow Card—MHRA in iOS

URL1: https://apps.apple.com/app/adr-online/id403478954, Accessed August 2020

URL2: https://play.google.com/store/apps/details?id=com.vinfotech.suspectedadversedrugreaction, Accessed August 2020

URL3: https://play.google.com/store/apps/details?id=com.amu.slidingmenu, Accessed August 2020

URL4: https://play.google.com/store/apps/details?id=com.dgda.adr&hl=en, Accessed August 2020

URL5: https://play.google.com/store/apps/details?id=be.intotheweb.easypharm, Accessed August 2020

URL6: https://apps.apple.com/app/easypharm/id1025013813, Accessed August 2020

URL7: https://play.google.com/store/apps/details?id=com.elea.oncobiotech&hl=en, Accessed August 2020

URL8: https://apps.apple.com/ch/app/elea-onco-biotech/id1328913431, Accessed August 2020

URL9: https://play.google.com/store/apps/details?id=hr.halmed, Accessed August 2020

URL10: https://apps.apple.com/app/halmed/id1080314179, Accessed August 2020

URL11: https://play.google.com/store/apps/details?id=com.epidemico.webradr, Accessed August 2020

URL12: https://apps.apple.com/gb/app/med-safety/id1439060917, Accessed August 2020

URL13: https://play.google.com/store/apps/details?id=com.GetGain.NationalPharmacovigilanceResearchCenter, Accessed August 2020

URL14: https://apps.apple.com/ru/app/%D0%BB%D0%B5%D0%BA%D0%B0%D1%80%D1%81%D1%82%D0%B2%D0%B5%D0%BD%D0%BD%D0%B0%D1%8F-%D0%B1%D0%B4%D0%B8%D1%82%D0%B5%D0%BB%D1%8C%D0%BD%D0%BE%D1%81%D1%82%D1%8C/id1487124395, Accessed August 2020

URL15: https://play.google.com/store/apps/details?id=fr.evedrug, Accessed August 2020

URL16: https://apps.apple.com/us/app/my-ereport/id806103319, Accessed August 2020

URL17: https://play.google.com/store/apps/details?id=com.baswedan.salamtok, Accessed August 2020

URL18: https://play.google.com/store/apps/details?id=siddha.drug.documentation, Accessed August 2020

URL19: https://play.google.com/store/apps/details?id=com.cive.HakikiDawaADR, Accessed August 2020

URL20: https://play.google.com/store/apps/details?id=com.mhra.mohap, Accessed August 2020

URL21: https://apps.apple.com/gb/app/uae-radr/id1374384487, Accessed August 2020

URL22: https://play.google.com/store/apps/details?id=uk.org.mhra.yellowcard, Accessed August 2020

URL23: https://apps.apple.com/app/yellow-card-mhra/id990237487, Accessed August 2020

The geographic specifications of the 22 selected apps are listed in Table 2. The highest number of apps were mapped for countries in the WHO African region (8/22), seven of which were Med Safety. These eight apps enabled users to report ADRs to the NRAs in Botswana, Burkina Faso, Côte d’Ivoire, Ethiopia, Ghana, Tanzania, Uganda and Zambia. Six apps were mapped for countries in the WHO European region, four in the WHO South-East Asia region, two in the WHO Eastern Mediterranean Region and one each in the WHO Region of the Americas and the WHO Western Pacific Region. In terms of the languages, English was used in most of the apps (20/22), while languages other than English, such as French, were also included in nine apps. Two apps from Argentina and the Russian Federation used only their own national language, respectively. When we classified the apps by country income level, as defined by the World Bank [33], more than 70% of the selected apps were in LMICs.

**Table 2:** Geographic specifications of the selected apps

App number	App name	Available languages	Country of data addressee	WHO region	World Bank country classifications by income level^d^	Report addressee
1	ADR Online	English	New Zealand	WPRO	High-Income Economies	New Zealand Pharmacovigilance Centre (NZPhvC)
2	ADR PvPI	English	India	SEARO	Lower-Middle-Income Economies	Pharmacovigilance Program of India (PvPI)
3	ADR Reporter	English	India^b^	SEARO	Lower-Middle-Income Economies	Aligarh Muslim University^b^
4	DGDA Drug Verification	Bengali, English	Bangladesh	SEARO	Lower-Middle-Income Economies	Directorate General of Drug Administration (DGDA) under the Ministry of Health & Family Welfare
5	Easypharm	Dutch, English	Belgium, Luxembourg	EURO	High-Income Economies	Federal agencies for medicines and health products, patient’s pharmacists
6	ELEA Onco-Biotech	Spanish	Argentina	PAHO	Upper-Middle-Income Economies	Laboratory Elea
7	HALMED	Croatian, English	Croatia	EURO	High-Income Economies	Agency for Medicinal Products and Medical Devices of Croatia (HALMED)
8	Med Safety (Armenia)	Armenian, English, Russian	Armenia	EURO	Upper-Middle-Income Economies	Scientific Center of Drug and Medical Technology Expertise (SCDMTE)
9	Med Safety (Botswana)	English	Botswana	AFRO	Upper-Middle-Income Economies	Botswana Medicines Regulatory Authority (BoMRA)
10	Med Safety (Burkina Faso)	English, French	Burkina Faso	AFRO	Low-Income Economies	National Pharmaceutical Regulatory Agency (ANRP)
11	Med Safety (Côte d’Ivoire)	English, French	Côte d’Ivoire	AFRO	Lower-Middle-Income Economies	Ivorian Pharmaceutical Regulation Authority (AIRP)
12	Med Safety (Ethiopia)	English	Ethiopia	AFRO	Low-Income Economies	Ethiopian Food and Drug Administration (EFDA)
13	Med Safety (Ghana)	English	Ghana	AFRO	Lower-Middle-Income Economies	Ghana Food and Drugs Authority
14	Med Safety (Uganda)	English	Uganda	AFRO	Low-Income Economies	Uganda National Drug Authority
15	Med Safety (Zambia)	English	Zambia	AFRO	Lower-Middle-Income Economies	Zambia Medicines Regulatory Authority (ZAMRA)
16		Russian	Russian Federation	EURO	Upper-Middle-Income Economies	National Pharmacovigilance Research Center
17	My eReport	Czech, Dutch, English, French, German, Italian, Portuguese, Romanian, Spanish	All European Union countries	EURO	High-Income Economies except Bulgaria which was classified as Upper-Middle-Income Economy	Countries’ authorities and industries
18	^c^	Arabic, English	Yemen	EMRO	Low-Income Economies	Yemeni Pharmacovigilance Center
19	SiddAR	English	India	SEARO	Lower-Middle-Income Economies	Pharmacovigilance Program of India (PvPI)
20	TMDA Adverse Reactions Reporting Tool	Swahili, English (partially available)	Tanzania	AFRO	Lower-Middle-Income Economies	Tanzania Medicines & Medical Devices Authority (TMDA)
21	UAE RADR	English	United Arab Emirates	EMRO	High-Income Economies	Ministry of Health and Prevention
22	Yellow Card Scheme^a^	English	United Kingdom of Great Britain and Northern Ireland	EURO	High-Income Economies	Medicines and Healthcare products Regulatory Agency (MHRA)

AFRO: African Region. EMRO: Eastern Mediterranean Region. EURO: European Region. PAHO: Region of the Americas. SEARO: South-East Asia Region. WPRO: Western Pacific Region

^a^The same app was named Yellow Card—MHRA in iOS

^b^ADR Reporter offered various methods for sending the ADR report such as email (gmail) where Aligarh Muslim University in India (which the developer was affiliated to) was set as the default addressee. The geographic scope and the addressees could be extended upon the entry of necessary information by the reporter

^c^Salamtok shared a report created in the app as an email (gmail), where the Yemeni Pharmacovigilance Center was set as the default addressee

^d^The World Bank Classifications were referred to [33]

#### E2B data elements

The E2B data elements that were available in the selected 22 apps are listed in Table 3. With the exception of one app (Salamtok, developed for reporting in Yemen), where only limited free-text fields were available, all apps consisted of E2B data elements, ensuring that at least the minimum required information could be collected. Patient name or initials were the most commonly adopted fields (21/22) for patient identification, followed by fields related to age and gender (20/22). To identify the reporter, a field for the reporter’s name was included in most of the apps (20/22). A field to describe the adverse reaction or select the reaction term from a defined list was available in 21 of the apps, of which three did not have further fields, such as date of reaction onset, outcome of reaction and seriousness criteria of reaction. A field to describe or select drug names from a defined list was available in 21 of the apps, of which two did not have further fields to add further details about the suspect drug, such as dosage, indication and route of administration.

**Table 3:** E2B data elements available in the selected apps

App number	App name	Minimal information category			
		Identifiable patient	Identifiable reporter	Adverse event/reaction (or outcome)	Suspect or interacting drug
1	ADR Online	Age at time of onset of reaction/Date of birth Medical history^a^ Name or initials Sex	Email address Qualification Reporter’s name	Reaction (text input) Fatality Date of start of reaction Outcome of reactions at the time of last observation Severity	Drug name (text input) Date of start of drug Date of last administration Dosage Indication Route of administration
2	ADR PvPI	Age at time of onset of reaction/Date of birth Height Medical history Name or initials Sex Weight	Email address Qualification Reporter’s name	Reaction (text input) Date of start of reaction Date of end of reaction Outcome of reactions at the time of last observation Seriousness criteria at event level	Drug name (text input) Date of start of drug Date of last administration Dosage Indication Route of administration
3	ADR Reporter	Age at time of onset of reaction/Date of birth Name or initials Sex	Email address Reporter’s name	Reaction (text input)	Drug name (text input) Dosage Indication Route of administration
4	DGDA Drug Verification	Age at time of onset of reaction/Date of birth Height Medical history Name or initials Sex Weight	Email address Qualification Reporter’s name	Reaction (text input) Date of start of reaction Date of end of reaction Outcome of reactions at the time of last observation Seriousness criteria at event level	Drug name (text input) Actions taken with drug Date of start of drug Date of last administration Dosage Indication Route of administration
5	Easypharm	Age at time of onset of reaction/Date of birth Name or initials Sex	Reporter’s name	Reaction (text input)	Drug name (text input)
6	ELEA Onco-Biotech	Name or initials	Email address Reporter’s name	Reaction (list)^c^	Drug name (list)^b^
7	HALMED	Age at time of onset of reaction/Date of birth Height Medical history Name or initials Sex Weight	Email address Qualification Reporter’s name	Reaction (text input) Date of start of reaction Date of end of reaction Outcome of reactions at the time of last observation Seriousness criteria at event level	Drug name (list) Actions taken with drug Date of start of drug Date of last administration Dosage Indication Route of administration
8–15	Med Safety	Age at time of onset of reaction/Date of birth Height Medical history Name or initials Sex Weight	Email address Qualification Reporter’s name	Reaction (list and text input)^d^ Date of start of reaction Date of end of reaction Outcome of reactions at the time of last observation Seriousness criteria at event level	Drug name (list) Actions taken with drug Date of start of drug Date of last administration Dosage Indication Route of administration
16		Age at time of onset of reaction/Date of birth Height Medical history Name or initials Sex Weight	Email address Qualification Reporter’s name	Reaction (text input) Date of start of reaction Outcome of reactions at the time of last observation Seriousness criteria at event level	Drug name (text input) Actions taken with drug Dosage Indication Route of administration
17	My eReport	Age at time of onset of reaction/Date of birth Height Medical history Name or initials Sex Weight	Email address Qualification Reporter’s name	Reaction (text input) Date of start of reaction Outcome of reactions at the time of last observation Seriousness criteria at event level	Drug name (list) Date of start of drug Dosage Route of administration
18		Only available free-text fields were labeled “City” and “Description”	Only available free-text fields were labeled “City” and “Description”	Only available free-text fields were labeled “City” and “Description”	Only available free-text fields were labeled “City” and “Description”
19	SiddAR	Age at time of onset of reaction/Date of birth Medical history Name or initials Sex Weight	Email address Qualification Reporter’s name	Reaction (text input) Date of start of reaction Date of end of reaction Seriousness criteria at event level	Drug name (text input) Date of start of drug Date of last administration Dosage Indication Route of administration
20	TMDA Adverse Reactions Reporting Tool	Age at time of onset of reaction/Date of birth Medical history Name or initials Sex Weight	Qualification	Reaction (text input) Date of start of reaction Date of end of reaction Outcome of reactions at the time of last observation Seriousness criteria at event level	Drug name (list and text input) Actions taken with drug Date of start of drug Date of last administration Dosage Indication Route of administration
21	UAE RADR	Age at time of onset of reaction/Date of birth Height Medical history Name or initials Sex Weight	Email address Qualification Reporter’s name	Reaction (list and text input) Date of start of reaction Date of end of reaction Outcome of reactions at the time of last observation Seriousness criteria at event level	Drug name (list) Actions taken with drug Date of start of drug Date of last administration Dosage Indication Route of administration
22	Yellow Card Scheme^e^	Age at time of onset of reaction/Date of birth Height Medical history Name or initials Sex Weight	Email address Qualification Reporter’s name	Reaction (list and text input) Date of start of reaction Date of end of reaction Outcome of reactions at the time of last observation Seriousness criteria at event level	Drug name (list) Actions taken with drug Date of start of drug Date of last administration Dosage Indication Route of administration

^a^Conditions available for selection: allergies, liver problems, kidney problems, other medical conditions, works with industrial chemicals, alternative medicines, nutritional supplements, over-the-counter medicines

^b^Available choices were limited to Bevax, Cimaher, Novex, Vaxira, Heberprot-P

^c^Available choices were limited to lack of effectiveness, adverse event, others

^d^Only text input was available in the Armenia and Burkina Faso versions

^e^The same app was named Yellow Card—MHRA in iOS

#### Additional apps features

**Fig. 2:**
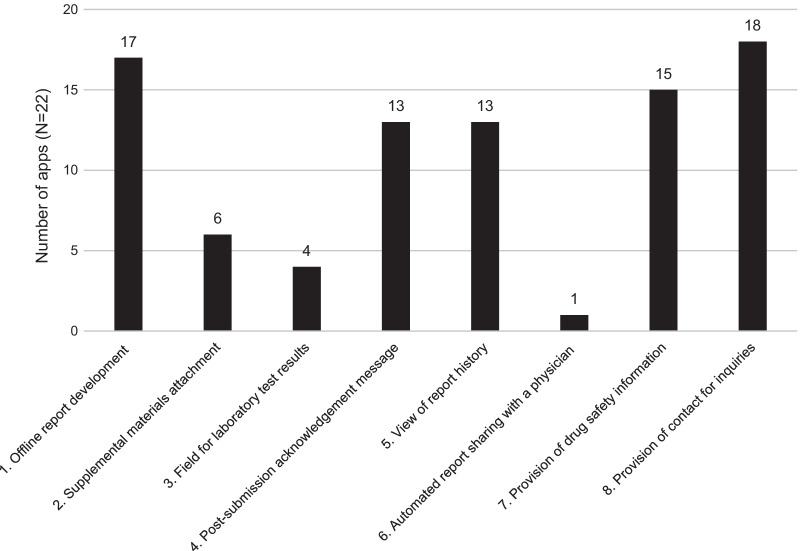
Number of apps offering each feature. Feature item No. 1 was present in the following apps: 1, 3, 6, 7, 8, 9, 10, 11, 12, 13, 14, 15, 18, 19, 20, 21, 22. Feature item No. 2 was present in the following apps: 1, 2, 17, 18, 19, 20. Feature item No. 3 was present in the following apps: 2, 4, 19, 20 (While not having a laboratory field by default, there were apps which automatically displayed supplementary fields and questions in response to an answer to certain conditions such as pregnancy). Feature item No. 4 was present in the following apps: 2, 7, 8, 9, 10, 11, 12, 13, 14, 15, 17, 21, 22. Feature item No. 5 was present in the following apps: 1, 7, 8, 9, 10, 11, 12, 13, 14, 15, 17, 21, 22. Feature item No. 6 was present in the following apps: 17. Feature item No. 7 was present in the following apps: 4, 6, 7, 8, 9, 10, 11, 12, 13, 14, 15, 16, 17, 21, 22. Feature item No. 8 was present in the following apps: 1, 3, 5, 7, 8, 9, 10, 11, 12, 13, 14, 15, 16, 17, 18, 19, 21, 22 (Apps were also counted if contact was included in an acknowledgment message or in an introduced website)

The available features that each selected app offered are shown in Fig. 2. The features that contributed to ADR reporting included providing support to create a report, report management, and two-way communication, such as not only enabling users to report but also providing drug safety information. Most of the apps enhanced reporting and two-way communication through a feature for offline report development (17/22), provision of contact addresses for inquiries (18/22) and provision of drug safety information (15/22). Apps rarely had an automated feature to allow a reporter to share the report with their physician or other addressees of their choice (1/22).

### Online Survey

The developers and owners of the selected 22 apps were invited to share their experiences of the apps through the online survey. Answers for 17 of the 22 apps (77.2%) from 19 interviewees were collected in November and December 2020. The answers were provided from No responses were received from the following five apps: ADR PvPI, Easypharm, Medicinal Vigilance, Salamtok and TMDA Adverse Reactions Reporting Tool.

All of the apps were launched after 2014 except ADR Online (developed for reporting in New Zealand), which was launched in 2010. The respondents confirmed that all the apps were developed to offer an additional tool for ADR reporting besides conventional methods. It was stated that the apps’ implementation was also intended to provide users with drug safety information and to raise awareness of ADR reporting through the engagement of a larger reporting population. The most frequent user types who reported ADRs through the apps were HCPs, such as pharmacists (5/17), medical doctors (4/17) and other HCPs (4/17). Following HCPs, patients or family members were reported as the next most frequent reporter type (3/17). Public health programs were not cited as frequent users in any of the responses.

#### Quantitative and qualitative impact of app implementation on ADR reporting

The quantitative data of app downloads and ADR reports are summarized in Table 4. The number of downloads tended to be higher for Android users compared to iOS, where both Android and iOS platforms were available. The apps which enabled users to report ADRs to public entities such as NRAs tended to have the higher number of downloads compared to the apps addressing ADRs only to private entities. The average percentage of ADR reports received from the apps compared to reports using all reporting methods varied from 0 to 60%. This range was broader for LMICs (0% to 60%) than for HICs (0% to 5%). A percentage greater than or equal to 5% was reported for eight apps: seven from LMICs and one from HICs.

**Table 4:** Quantitative impact of app implementation for the apps cited in the online survey

App number	App name (country of data addressee)	App launch date	Data provided by developers and owners of the selected apps				
			Number of downloads (Android) since the launch^b^	Number of downloads (iOS) since the launch^b^	Trend of overall number of ADR reports since the launch	Number of ADR reports received from the app since the launch (average number per year)	Average % of ADR reports received from the app among all reports since the launch
1	ADR Online (New Zealand)	1 November 2010	App not available on the platform	10,001–50,000	No impact	~ 100 (~ 9.1)	0.2%
3	ADR Reporter (India)	16 October 2016	101–1000	App not available on the platform	Unknown	n/a (n/a)	n/a
4	DGDA Drug Verification (Bangladesh)	30 June 2019	10,001–50,000	App not available on the platform	Increased	79 (55.6)	3–5%
6	ELEA Onco-Biotech (Argentina)	22 December 2017	1–100	1–100	No impact	0 (0)	0%
7	HALMED (Croatia)	18 May 2016	1001–10,000	101–1000 from the launch to 29 October 2019	Increased	499 (109.9)	3%
8	Med Safety (Armenia)	7 May 2019	101–1000 from launch to 29 October 2019	101–1000 from launch to 29 October 2019	Increased	21 (13.3)	5%
9	Med Safety (Botswana)	14 November 2019	101–1000	1–100	Increased	32 (30.4)	9%
10	Med Safety (Burkina Faso)	15 June 2017	1001–10,000	1–100	Increased	350 (101.1)	40%
11	Med Safety (Côte d’Ivoire)	17 December 2019	101–1000 from February to November 2020^c^	No impact	10 (10.4)	20%	
12	Med Safety (Ethiopia)	23 August 2019	101–1000 from February to November 2020^c^	Increased	50 (39.3)	3%	
13	Med Safety (Ghana)	25 June 2019	1001–10,000	1001–10,000	No impact	113 (78.4)	4%
14	Med Safety (Uganda)	26 February 2020	1001–10,000 from February to November 2020^c^	No impact	140 (184.2)	5%	
15	Med Safety (Zambia)	29 June 2017	1001–10,000^c^	Increased	n/a (n/a)	~ 5%	
17	My eReport (All European Union countries)	6 February 2014	1001–10,000	1001–10,000	No impact	4221 (618.9)	5%
19	SiddAR (India)	6 March 2018	101–1000	App not available on the platform	Increased	10 -20 per month (120–240)	60%
21	UAE RADR (United Arab Emirates)	20 January 2019	101–1000	101–1000	No impact	7 (3.7)	0.09%
22	Yellow Card Scheme (UK)^a^	15 July 2015	1001–10,000	10,001–50,000	Increased	1000 + ; 40–60 per month (480–720)	~ 2.5%

n/a: Not available

^a^The same app was named Yellow Card—MHRA in iOS

^b^The period for which the data were available for the analysis is indicated. Otherwise, the period from the launch to data extraction was applied

^c^Only aggregated data including the number of downloads in Android and iOS were available

More than half of the respondents said that they had observed a post-launch upward trend in the overall number of ADR reports, not only for those received through the apps but also from other reporting tools, such as paper-based reporting (9/17).

**Fig. 3:**
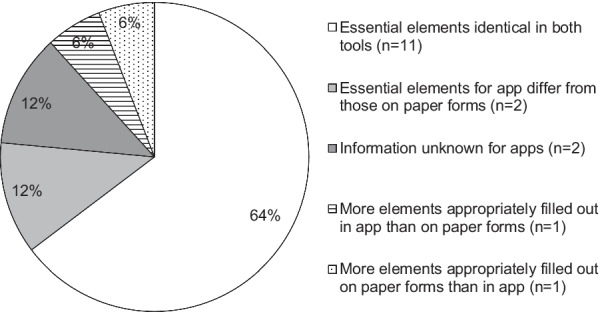
Comparison of essential elements* appropriately filled in for ADR reports on apps and in paper form. *Elements making up the “minimum information for valid safety report” as defined by the ICH

Figure 3 shows qualitative differences in “essential elements” appropriately filled in for ADR reports submitted from apps and in paper form. 65% of respondents (11/17) said that the number of elements appropriately filled-in on reports submitted via app was the same as that for paper reports. Six respondents said that all of the four minimum elements were appropriately filled-in on reports submitted using the apps and paper forms.

#### Real-world experiences with ADR reporting apps

The respondents of the online survey shared the pros and cons of their ADR reporting apps (Table 5). The inputs from the respondents were synthesized into the following themes:

**Table 5:** Respondents’ comments on use of ADR reporting apps

	Accepted features	Opportunities for further consideration and improvement
Simplicity of use	ADR reporting via app is simplified, easier and requires less time [than other reporting forms], contributing to the elimination of reporting delays Apps include functions that facilitate data entry, such as dropdown menus, lists of medicines, and a “save” option to continue reporting later Information can be further reported in detail through attachments Storage of apps requires a small amount of data memory Apps provide adequate space for case narrative compared to paper forms with limited space	App collects only limited ADR information and must be complemented by comprehensive reporting by an ADR monitoring centre App takes longer to fill in than paper forms. Difficult to fill in electronic forms on small mobile phone
Quality of reports	Apps eliminate quality issues such as missing reporter names and drug names due to mandatory fields	No comments made
Accessibility	Apps make ADR reporting more accessible to all segments of society, such as patients and healthcare professionals, as anyone with a smartphone can report Offline features allow users to use some functions even without an internet connection Translation of apps into local languages makes them more accessible	Healthcare professionals must sometimes download the app onto their personal phone, which they may deem inappropriate since the app is work-related App users need internet access to download and use the online functions Users may be reluctant to complete the full user registration process. Also, users may not have email addresses required for the registration
Innovativeness	Apps enable us to keep up with the contemporary digital world	Apps are mostly suitable for the younger generation and users with an inclination to use technology. Technology-averse or older populations may be reticent about using them
Data transferability and data sharing	ADR reports are received directly to NRAs in national ADR databases, which contributes to preventing the need for manual data entry, thus saving time. This also eliminates possible transcribing errors Apps are structured in the E2B format required for data processing	Apps do not allow reporters to save a copy of a report to refer back to once it has been sent and thus to share the report with multiple internal and external entities. This limits administrative reporting processes, which are routinely followed in some countries
Two-way communication	Apps provide users with a range of information, such as safety data on medicines and other related news, in a timely manner Apps engage an audience interested in the safety of medicinal products Immediate acknowledgement messages after report submissions are appreciated by users	If apps do not display contact information, users cannot get in touch with regulators or relevant bodies for clarifications or questions concerning the reporting Users may fail to read news if the apps do not have a notification feature for the news of interest Only a limited audience is likely to download apps for regulatory purpose
Costs	ADR reporting in free of charge for reporters (except the fee for data transfer) Cuts the cost of distributing reporting tools to the users	No comment made
Data security	No comments made	In apps that save reports until they are deleted by users, apps may have no control over patient information being uploaded to the Cloud if the device is backed up there Users may not want to provide details, such as their name, institution and contacts

### Simplicity of use and report quality

Respondents said that the apps simplified ADR reporting, making it quick and easy, and reducing delays in reporting to a data addressee such as a competent authority. The simplicity was enhanced by digital features, such as drop-down menus, a defined drug list, and data file attachment capability. Defining mandatory reporting fields reduced issues of missing data and increased the overall quality of reports. However, the limited information collected by such simple reporting apps needed to be complemented by more comprehensive reporting afterward.

### Innovativeness and accessibility of reporting

Feedback indicated that apps make ADR reporting accessible to a broader population since anyone with a smartphone can download the app and report ADRs. Although a feature enabled the use of some functions without an internet connection, some internet connectivity was needed later to upload the offline activity so it could be transmitted. Respondents in countries with limited internet connectivity reported persistent difficulties in using the apps. Respondents said the younger generation and/or users with an inclination to use technology were more likely to use apps compared to more technology-averse or older people.

### Data transferability and data sharing

NRAs valued the direct receipt of ADR reports from apps to their databases since it did not require the manual entry of data, thus saving time and potentially avoiding transcribing errors. Moreover, apps structured in the E2B format were convenient for data processing. A limitation was the inability of the app to save a copy of a report for the reporter to refer back to once it had been sent and to thus be able to share the report with other relevant stakeholders.

### Two-way communication

Two-way communication enabled owners and end users to communicate drug safety information and other related news in a timely manner, and strengthened the engagement of end users with an interest in drug safety. When end users received an immediate acknowledgment of their report’s submission, it bolstered their commitment by making them feel they were making a valuable contribution to pharmacovigilance activities.

### Costs

Respondents expressed only positive comments on cost since the apps were free of charge for reporters and reduced the cost of distributing paper reporting forms.

### Quantitative search on number of reports submitted to VigiBase before and after app launch

Of the 17 apps considered in this analysis, the full observational period of 12 months in post-launch Period A was available in 16 apps, and post-launch Period B in 10 apps. The apps that did not have a post-launch Period B were DGDA Drug Verification, Med Safety (Armenia), Med Safety (Botswana), Med Safety (Côte d’Ivoire), Med Safety (Ethiopia), Med Safety (Ghana) and Med Safety (Uganda), while Med Safety (Uganda) was not included in either analysis.

An upward trend in the number of ADR reports was observed in 81.2% of the apps (13/16) in post-launch Period A compared to the pre-launch period. The remaining three apps—ADR Reporter, Med Safety (Burkina Faso) and SiddAR—showed a negative trend. The relative change in post-launch Period A varied from 11.9% to 596.7% in the apps showing an upward trend. Only four apps showed a continuous upward trend over post-launch Periods A and B compared to the pre-launch period. Med Safety (Burkina Faso) was the only app to show an upward trend in post-launch Period B (+ 71.4%) following a downward trend in post-launch Period A (− 42.4%). Further details are shown in Table 6.

**Table 6:** Quantitative trend of ICSRs received in VigiBase over different pre- and post-launch periods for the apps cited in the online survey

App number	VigiBase data				
	App name (country of data addressee)	App launch date	Pre-launch period (Reference)	Post-launch Period A (Relative % change compared to Reference)	Post-launch Period B (Relative % change compared to Reference)
1	ADR Online (New Zealand)	1 November 2010	4704	6488 (+ 37.9%)	4332 (− 7.9%)
3	ADR Reporter (India)	16 October 2016	75,217	66,527 (− 11.6%)	52,085 (− 30.8%)
4	DGDA Drug Verification (Bangladesh)	30 June 2019	0	6 (n/a)	n/a (n/a)
6	ELEA Onco-Biotech (Argentina)	22 December 2017	509	987 (+ 93.9%)	9628 (+ 1791.6%)
7	HALMED (Croatia)	18 May 2016	3550	3972 (+ 11.9%)	4589 (+ 29.3%)
8	Med Safety (Armenia)	7 May 2019	240	638 (+ 165.8%)	n/a (n/a)
9	Med Safety (Botswana)	14 November 2019	38	134 (+ 252.6%)	n/a (n/a)
10	Med Safety (Burkina Faso)	15 June 2017	399	230 (− 42.4%)	684 (+ 71.4%)
11	Med Safety (Côte d’Ivoire)	17 December 2019	11	66 (+ 500%)	n/a (n/a)
12	Med Safety (Ethiopia)	23 August 2019	147	209 (+ 42.2%)	n/a (n/a)
13	Med Safety (Ghana)	25 June 2019	728	5072 (+ 596.7%)	n/a (n/a)
14	Med Safety (Uganda)	26 February 2020	1200	n/a (n/a)	n/a (n/a)
15	Med Safety (Zambia)	29 June 2017	36	112 (+ 211.1%)	0 (− 100%)
17	My eReport (All European Union countries)	6 February 2014	156,428	388,320 (+ 148.2%)	206,879 (+ 32.3%)
19	SiddAR (India)	6 March 2018	67,039	65,223 (− 2.7%)	64,422 (− 3.9%)
21	UAE RADR (United Arab Emirates)	20 January 2019	1634	3628 (+ 122.0%)	870 (− 46.8%)
22	Yellow Card Scheme (UK)^a^	15 July 2015	30,025	55,159 (+ 83.7%)	44,080 (+ 46.8%)

n/a: Not applicable. Pre-launch period: 12-month period preceding the app’s launch; Post-launch Period A: Period from launch month to Month 12; Post-launch Period B: Period from Month 13 to 24

^a^The same app was named Yellow Card—MHRA in iOS

## Discussion

To our knowledge, this is the first study mapping ADR reporting apps. Of the 22 apps selected for the study, more than 70% were based in LMICs, with the majority in the WHO Africa region.

Although the number of LMICs with pharmacovigilance centers reporting to VigiBase has increased substantially in the last 10 to 15 years, actual reporting per 100,000 population per year is relatively low in LMICs compared to many HICs, which have well-established and more mature pharmacovigilance systems [34–36]. Paper-based reporting requires reports to be manually transported by postal services to a pharmacovigilance center once filled out by a reporter, and to be manually entered into a data management system, which is one of the most time-consuming activities in the reporting process [37]. It is likely to result in quantitative and temporal challenges, i.e. delay from the time of the onset of an ADR to the time the report reaches VigiBase [38]. Moreover, LMICs face qualitative challenges due to poorly documented reports [39]. Needless to say, the comprehensiveness of a report plays a critical role in pharmacovigilance in order to aid in the detection of signals [40].

This study showed that ADR reporting apps offer unique added value compared to conventional reporting tools, such as paper-based formats, through various features, and they were likely to support countries to more efficiently collect data. One of the identified features was a standardized format meeting E2B standards [41]. Hence data collected by the apps were easily transferable to the E2B-compatible national data management system and, moreover, the flow of data to VigiBase would be facilitated, thereby addressing the challenges that LMICs face. In fact, according to the online survey results, LMICs were more likely than HICs to take advantage of smartphone-based apps features for reporting. The survey respondents also appreciated that the apps supported data transmission and improved report quality in LMICs. However, not all respondents were able to provide information to thoroughly describe the direct impact of the apps. Further investigation would be warranted on outcomes, such as the time from event onset to data submission on VigiBase, and the report quality appraised through various measurements [39, 40, 42] between those received from ADR reporting apps and those via conventional routes.

Notably, in this study, more than 50% of the survey respondents commented that apps contributed to an increase in the number of *all* ADR reports regardless of the type of reporting tool. The upward quantitative trend of all ICSRs in the post-launch periods was also confirmed by the VigiBase search. As mentioned in the introduction, several countries have been supported by the WHO to roll out the ADR reporting app, Med Safety. The first country to pilot the app was Burkina Faso in 2017 [43, 44], initially as part of the seasonal malaria chemoprevention campaign. Promotional campaigning and trainings were integrated into app roll-out as part of a national malaria disease program. Along with the launch, an extensive campaign was also conducted in Uganda, including a month-long intensive mass media campaign through television, radio, local newspapers and a press conference. The launch attracted hundreds of stakeholders in pharmacovigilance [45]. ADR reporting tools based on user-friendly technology could be appealing to the public and strengthening the culture of reporting. Moreover, integration of pharmacovigilance into the work of other relevant partners, such as public health programs, should have been an indispensable contributor to successful app implementation, and might have indirectly resulted in a rise in the overall number of reports.

Although offline app function was cited as a positive driver in influencing patients and HCPs to use the ADR reporting app in the Innovative Medicines Initiative (IMI) WEB-Recognizing Adverse Drug Reactions (RADR) project [46, 47], this study reported persistent difficulties due to internet connectivity issues in some geographical settings. In this survey, this challenge was reported particularly by LMICs and given as one of the reasons for the low use of the app despite the offline function. Although it was reported that there was a high coverage of fourth generation broadband cellular network technology (4G) in LMICs (82% of the population), it is also known that such services are not affordable in more than half of LMICs [48, 49]. In addition, internet stability is a challenge due to unexpected technical issues and sporadic political restrictions [50, 51]. App use could also be influenced by individual socio-demographic status and level of interest [46], and is preferred by the younger generation and those with an interest in the technology. Additional barriers exist in LMICs due to rural–urban differences and gender gaps in mobile internet use, as well as a lack of literacy and digital skills [48]. It may be important to consider the level of digital literacy required to master the settings when introducing an app, to identify additional follow-on support, such as training, that might be required to ensure optimal app implementation and use.

The overall benefit of the apps should be examined alongside the financial affordability of the technology. Conventional paper reporting incurs various production costs, such as for paper, printing, distribution and postage. In addition to these direct costs, the indirect costs related to the human resources, time and effort required to manually process each paper form received would not be negligible [37]. The cost saving on these items that the apps offer was positively appraised in our online survey. Nevertheless, apps incur their own costs throughout implementation, e.g. pre-development research, design, technical development, testing, deployment, and continual support and maintenance [52]. To ensure that NRAs make cost-effective decisions, we highlight the importance of considering the cost as well as the benefits of an app when deciding whether to develop and implement it.

Although this study presents globally generalizable evidence based on a robust systematic methodology, the results should be cautiously interpreted due to their limitations. Firstly, although the number of hits from the searches of the Google Play Store and App Store varied depending on the search terms, their seemed to be a ceiling of about 250 items in the displayable hit numbers, especially in the Google Play Store. As a result, it may be possible that some apps did not appear in our searches. Secondly, we ran the searches using English terms, possibly excluding some of the non-English apps, although apps available only in languages other than English were also picked up by our search strategy and included in the study. Also, searches were performed from a single location (Geneva, Switzerland) and the results may differ from another search location. A supplemental study using search terms translated into various languages, such as the official United Nations languages, and performed in different locations worldwide would be worthwhile. Lastly, information technology is advancing rapidly. The selected apps will no doubt be updated and the latest versions currently in use might not be identical to the ones reviewed in this study. Also, additional apps may have been developed in the interim. It would be useful to repeat this survey periodically, to capture innovations and new pharmacovigilance apps.

## Conclusions

There are various kinds of ADR reporting tools. App-based ADR reporting tools are becoming more popular in different regions of the world and they contribute to ADR reporting through technological features. They also strengthen the overall culture of ADR reporting in their appeal to a wider group of reporters and due to the ease of reporting. ADR reporting apps have the potential to support pharmacovigilance activity; however, in launching an app, it is important to consider features and functions that can contribute to a qualitative and quantitative improvement in reporting, and considering the proposed user group, any training needs, and the costs involved to develop, launch and maintain the app. Moreover, further post-implementation studies would help assess the long-term impact of app-based tools and how these can be sustained.

## Data Availability

The authors confirm that the data supporting the findings of this study are available within the article and its supplementary materials.

## Appendix 1: Search terms used in App Store and Google Play Store

**Table:**

1. ‘adverse event’ 2. ‘ADR drug’ 3. ‘ADR reporting’ 4. ‘adverse drug event’ 5. ‘adverse drug reaction’ 6. ‘adverse effect’ 7. ‘adverse outcome’ 8. ‘adverse reaction’ 9. ‘drug interaction’ 10. ‘drug safety report’ 11. ‘drug surveillance’ 12. ‘drug toxicity’ 13. ‘medication error’ 14. ‘medicine safety’ 15. ‘patient safety’ 16. ‘pharmacovigilance’ 17. ‘side effect’	

## Appendix 2: List of questions asked in the online survey

**Table:**

1. On which date was the app launched? 2. On which date was the app last updated? 3. What was the purpose of the app’s development/implementation? 4. To which countries does the app allow users to submit ADR reports? 5. What was the total number of downloads of the app from the iOS platform since the app’s launch? a. 1–100 b. 101–1000 c. 1001–10,000 d. 10,001–50,000 e. 50,001 or more f. App is not available on the iOS platform g. Unknown 6. What is the total number of downloads of the app from the Android platform since the app’s launch? a. 1–100 b. 101–1000 c. 1001–10,000 d. 10,001–50,000 e. 50,001 or more f. App is not available on the Android platform g. Unknown 7. Did you notice an upward or downward trend in the number of downloads of the app per month in 2020 compared to 2019? a. Upward trend b. Downward trend c. No change d. Unknown 8. How has the app’s implementation impacted the overall number of ADR reports that you receive? a. The app’s implementation has increased the number of ADR reports b. The app’s implementation has decreased the number of ADR reports c. The app’s implementation has not impacted the number of ADR reports d. Unknown e. Other 9. How many ADR reports have been received by the app since its launch? 10. What is the proportion of ADR reports received from the app among all reports received since the app’s launch? (%) 11. What was the most frequent reporter type using the app for ADR reporting? a. Medical doctor b. Pharmacist c. Other healthcare professional d. Patient or family member e. Public health program f. Unknown g. Other 12. In general, among the following essential elements*, which were appropriately filled-in on the ADR reports received by the app? (Check all relevant answers.) a. Patient b. Reporter c. Product exposure d. Event e. The information is unknown 13. In general, among the following essential elements*, which were appropriately filled-in on the ADR reports received on a paper reporting form? (Check all relevant answers.) a. Patient b. Reporter c. Product exposure d. Event e. The information is unknown 14. If you have any further comments on the quality and quantity of ADR reports received by the app compared to reports received through other tools, please add these below 15. What was the total cost of app development? Please enter an amount in the appropriate currency. If unknown, please say ‘Unknown’ 16. What is the annual cost of app maintenance? Please enter an amount in the appropriate currency. If unknown, please say ‘Unknown’ 17. What are the funding sources for these costs? If unknown, please say ‘Unknown’ 18. Please share your experiences of the development of the app 19. What are the pros and cons of the app?	

*Elements making up the “minimum information for valid safety report” as defined by the ICH.

## Abbreviations

ADR: Adverse Drug Reaction
ICH: International Council for Harmonisation of Technical Requirements for Pharmaceuticals for Human Use
IMI: Innovative Medicines Initiative
MHRA: Medicines and Healthcare products Regulatory Agency
NRA: National Regulatory Authority
PRISMA: Preferred Reporting Items for Systematic reviews and Meta-Analyses
UMC: Uppsala Monitoring Centre
WEB-RADR: Web-Recognizing Adverse Drug Reactions
WHO: World Health Organization

## Footnotes

1 The app and its description were listed on the app store, but it could not be found when we sought to download it.

## Declarations

### Declarations

Ethics approval and consent to participate.

Not applicable.

Consent for publication.

Not applicable.
